# Stakeholder perspectives on simulation-based education in radiation oncology: opportunities and barriers^☆^

**DOI:** 10.1016/j.tipsro.2026.100448

**Published:** 2026-10-23

**Authors:** M. Kok, M.E.M. Den Ouden, I.M. Hertel, J.P.M. van Bergen, I. ten Haken, C. Jaschinski

**Affiliations:** a Department of Radiation Oncology, Radboudumc Nijmegen, the Netherlands; b Research Group Technology, Health & Care, School of Social Work, Saxion University of Applied Sciences, the Netherlands

**Keywords:** Simulation-based education, Work-based learning, Radiation Oncology, RTT education, Stakeholder perspectives

## Abstract

Radiation therapists (RTTs) face increasing clinical demands, limiting opportunities for structured training and feedback during work-based learning. This study explored whether simulation-based education (SBE) could address these challenges. Semi-structured interviews were conducted with 16 stakeholders (i.e. students, RTTs, managers and educators) in a Dutch academic hospital. Participants valued SBE for providing a safe environment for repetitive practice, feedback and bridging theory and practice. Reported barriers included time, costs, resources and limited fidelity. Technical skills, methodical working and teamwork were seen as suitable learning objectives. The study concludes that successful SBE implementation requires institutional support, trained faculty and realistic, learner-centred scenarios.

## Highlights

• The development and implementation of Simulation Based Education (SBE) requires multistakeholder collaboration.
• Stakeholder collaboration should result in shared understanding, ambitions and informed decision-making on implementation.
• SBE enables repetitive practice in safe, realistic learning environments.
• Successful SBE implementation requires time, institutional support, financial resources and faculty training.

## Introduction

With an aging population, increasing cancer incidence and growing care complexity, healthcare systems worldwide are under substantial pressure. Radiation therapists (RTTs) are expected to deliver high-quality safe care while also supervising students in work-based learning. However, staffing constraints and high clinical workloads limit opportunities for structured training, feedback and professional development for both students and RTTs [1], [2].

In the Netherlands, RTTs are primarily trained through bachelor's degree programs in Medical Imaging and Radiation Therapy, which combine theoretical education with clinical placements. In-service training pathways are also available. Following graduation, professional development continues through work-based learning and department-specific training. Within this educational framework, work-based learning plays a central role in the development of professional competencies. Although simulation-based education (SBE) is increasingly used in healthcare education, its integration into radiotherapy work-based learning remains limited and varies across institutions.

SBE is a promising approach to strengthen work-based learning by creating authentic yet safe learning environments that mirror clinical practice [3], [4]. SBE enables repeated practice, structured skill development, feedback and reflection without compromising patient care [4], [5]. Previous studies in healthcare show that both students and clinical professionals perceive SBE as valuable, mainly as a supplement to hands-on clinical experience rather than a replacement [6], [7]. Most studies focus on student outcomes, such as skill acquisition, confidence and preparedness [7], [8]. Fewer studies have examined how educators, clinical staff and managers perceive SBE or how it can be effectively integrated into routine work-based learning. Understanding these perspectives is critical to ensure that SBE is relevant, feasible and aligns with clinical practice and departmental resources. Hence, this study explores the perspectives of students, clinical staff, educators and managers on the integration of SBE into work-based learning for RTTs, focusing on suitable learning objectives, opportunities and barriers for future implementation in clinical practice.

## Materials and methods

### Study design and setting

An exploratory qualitative study was conducted within the Radiation Oncology department of a Dutch academic hospital. The aim of this study was to explore stakeholder perspectives on integrating SBE into work-based learning for RTTs.

### Participants and sampling

A purposive sample of stakeholders involved in RTT education and clinical supervision was recruited. In total, 16 participants were included: RTT students (*n* = 2), early-career RTTs (<3 years' experience, *n* = 5), experienced RTTs (≥3 years' experience, *n* = 6), team leaders (*n* = 1), tactical managers (*n* = 1) and an educator from a collaborating educational institution (*n* = 1). RTTs were the largest group, reflecting their central role in work-based supervision. Participant characteristics are summarized in Table 1.

**Table 1.** Participant characteristics (*n* = 16).

Characteristics		Value
Gender	Female, n (%)	13 (81%)
	Male, n (%)	3 (19%)
Age, mean (range)		35.3 (21–61) years
Background	Students	2
	Early-career RTTs (<3 years)	5
	Experienced RTTs (≥3 years)	6
	Team leader	1
	Tactical manager	1
	Educator	1

Participants were recruited through voluntary responses to internal communication and through personal invitations of managerial and educational stakeholders. Participants were eligible if they belonged to one of the predefined stakeholder groups involved in RTT education, clinical supervision, management or training. Prior experience with SBE was not required, as the study aimed to capture a broad range of perspectives regarding its potential integration into work-based learning. No additional inclusion or exclusion criteria were applied.

### Data collection

Semi-structured interviews were conducted between February and March 2025, either face-to-face or online via videoconferencing. Interviews lasted 40–60 min and were audio-recorded. Recordings were transcribed verbatim using the transcription function in Microsoft Word and subsequently checked and corrected against the original recordings by the first author before qualitative analysis. The semi-structured interview guide was adapted from an existing stakeholder interview framework developed for a Dutch National Growth Fund project on SBE and contextualized for radiation therapy [9]. The guide allowed flexibility to explore topics raised by participants while ensuring consistency across the predefined interview domains. Questions addressed three domains: (1) current work-based learning practices, (2) experiences with and perceptions of SBE and (3) opportunities, barriers and conditions for future implementation. To stimulate reflection, participants were presented with a short future-oriented scenario describing SBE as an integrated component of work-based learning. This scenario facilitated discussion of its practical feasibility and anticipated impact.

### Data analysis

Transcripts were analysed using thematic analysis [10] in ATLAS.ti (version 25). An initial deductive coding framework was developed based on the interview domains and relevant literature. Following initial coding, codes were reviewed, compared and grouped into overarching themes. Themes were subsequently refined to ensure alignment with the coded data. To enhance trustworthiness, a second researcher independently coded three interviews. Coding discrepancies were discussed until consensus was reached, resulting in minor refinements to code definitions.

### Ethical considerations

Participants provided informed consent prior to participation and all data were handled confidentially. No ethics approval was applied as this study was not subject to the Medical Research Involving Human Subjects Act, participants were not asked to act or to change behaviors and the questions were not of a drastic nature.

## Results

### Current work-based learning and education

Respondents described the educational pathways represented in this study as largely practice-oriented, using formative assessment and programmatic testing to support progressive competency development. Some SBE is already used through the Virtual Radiation Therapy simulator (VERT), role-playing and simulated patients to support communication and teamwork skills. The learning environment was generally perceived as supportive and well structured, with motivated staff supervising students.

However, respondents noted that limited theoretical hours and high clinical workload restrict opportunities for deeper learning and clinical reasoning. Students and RTTs with less than three years of experience also perceived an overemphasis on reflection, feedback and report writing, while practical performance in real clinical settings received insufficient attention. Resource limitations and the limited realism of the VERT were further mentioned as barriers.

### Current knowledge and attitudes toward simulation-based education

Most respondents were familiar with SBE, typically describing it as role-playing or scenario-based simulation. However, their knowledge of different forms of SBE varied across stakeholder groups. Students and RTTs with less than three years of experience mainly knew VERT, skills labs, role-playing and simulated patients, while RTTs with more than three years of experience also mentioned virtual reality, e-learning and screen-based simulations. Educational and managerial stakeholders reported familiarity with a broad range of approaches, including serious gaming, virtual reality, role-playing and e-learning applications.

Overall, attitudes toward SBE were predominantly positive. Respondents viewed SBE as a valuable complement to work-based learning, supporting both student development and ongoing professional training. All respondents expressed willingness to adopt SBE in their educational or clinical practice.

### Opportunities for simulation-based education

Several opportunities for SBE were identified. SBE enables repetitive practice in a safe, controlled environment, without compromising patient care. Respondents emphasized the value of making mistakes without consequences and receiving immediate feedback, which enhances the learning process. This opportunity was recognized across all stakeholder groups. Respondents valued the ability to create realistic and varied scenarios, including complex, infrequent or time-pressured situations that are difficult to encounter during routine clinical work. Students particularly highlighted the value of practicing complete treatment pathways, emergency situations and complex cases, whereas RTTs emphasized opportunities to rehearse workflows and infrequently encountered clinical scenarios.

SBE was also viewed as flexible and adaptable, supporting individualized learning at one's own pace. Early skill development, such as becoming familiar with equipment and procedures before entering clinical practice, was considered beneficial for both students and experienced professionals. The team leader and tactical manager additionally highlighted the potential of SBE to support familiarization with new equipment, procedures and specializations.

SBE was perceived to help students feel more prepared for clinical practice by reducing the gap between theory and practice. By simulating real-world situations, students develop a clearer understanding of the clinical environment, making their work-based learning more effective. Finally, SBE was considered to reduce pressure on clinical capacity by shifting part of the training outside the clinical environment.

### Barriers to simulation-based education

Several barriers to SBE were identified. The main barrier was that simulations cannot fully replicate real-life clinical situations, particularly the complexity of interacting with diverse patients. RTTs and the tactical manager specifically noted that communication with real patients is difficult to simulate and remains an essential aspect of radiotherapy practice. As a result, respondents viewed SBE as a useful complement but not a substitute for hands-on clinical practice. Practical and organizational barriers were also noted, including the high development costs, the time and resource required to develop and run simulations and technological dependence with the risk that simulations may quickly become outdated. Finally, personal barriers were mentioned, such as cybersickness (e.g., headaches or dizziness) when using VR equipment and the perception that simulations may feel artificial or uncomfortable. In addition, students may overestimate their competencies in simulated environments, leading to gaps in actual clinical performance.

### Suitable learning objectives for simulation-based education

All respondent groups considered radiotherapeutic procedures well suited for SBE. This included task-specific skills, such as operating equipment, understanding its functions, following treatment workflows and positioning patients. Simulation was also valued for supporting methodical, step-by-step practice with VR and mixed reality seen as effective tools for these purposes.

Moreover, students, RTTs and the educator emphasized the value of simulation for practicing teamwork and collaboration during patient positioning and other procedures, offering opportunities to practice coordination, communication and social skills within the team. Clinical reasoning and problem-solving were also considered suitable for simulation, particularly by students, RTTs, the educator and the team leader.

Simulation was seen as a way to practice decision-making in scenarios with multiple possible approaches and to work under time constraints. These skills could be practiced using physical simulations such as skills labs, role-plays, simulated patients, or via mixed reality. Simulation was also seen as useful for department-specific procedures and specialties. RTTs and the team leader highlighted its potential to support onboarding within department-specific workflows and familiarization with new equipment.

However, some elements were considered difficult to simulate, including patient-dependent skills, ad hoc organizational tasks, administrative work and situations shaped by workload or unexpected deviations from planned procedures.

### Conclusion and discussion

This study shows that SBE is a relevant, future-oriented approach for strengthening work-based learning in radiotherapy, with clear potential to address educational challenges. These findings are supported by broad stakeholder engagement, indicating its relevance for radiotherapy education and practice. Importantly, SBE can replace some elements of work-based learning, however particularly patient-dependent skills such as communication with patients or responding to diverse patient needs are difficult to train only using SBE. The findings are consistent with existing literature, which highlights the value of simulation for safe, repetitive practice and skill development, while also emphasizing that simulation cannot fully replicate real clinical environments [11], [12]. Differences in perceptions regarding what can be effectively simulated may reflect varying levels of familiarity with SBE, underlining the importance of adequate training and guidance across stakeholder groups. Successful implementation of SBE also depends on the involvement of educators, RTTs involved in workplace supervision, team leaders and managers. These stakeholders contribute to identifying suitable learning objectives, facilitating learning activities and ensuring alignment between simulation activities and clinical practice. Their involvement may therefore support the effective integration of SBE into radiotherapy work-based learning. When thoughtfully integrated, SBE has the potential to enhance preparedness for practice, support professional development and contribute to sustainable workforce development in radiotherapy.

While several opportunities and barriers identified in this study are consistent with broader simulation literature [11], [13], [14], our findings extend existing knowledge by demonstrating how stakeholders perceive the role of SBE within radiotherapy work-based learning. Beyond supporting student learning, participants highlighted applications for onboarding newly qualified RTTs, training within department-specific procedures and specializations, and maintaining competencies for infrequently performed tasks. These applications are particularly relevant within radiotherapy, where professionals work with complex technologies, standardized treatment workflows and continuously evolving equipment and procedures.

Together, these findings suggest that the role of SBE in radiotherapy may extend beyond undergraduate education to include onboarding, department-specific training and ongoing professional development.

Our findings suggest that involving multiple stakeholder groups may facilitate the development and implementation of SBE within radiotherapy work-based learning. Stakeholders identified several factors that may influence successful implementation, including institutional support, sufficient time, available resources and faculty training. Participants also emphasized the importance of realistic simulations that are aligned with clinical practice and tailored to learners' needs. These findings provide practical considerations for departments exploring the integration of SBE and may help guide future implementation efforts in radiotherapy education and practice.

## Declaration of generative AI and AI-assisted technologies in the manuscript preparation process

Statement: During the preparation of this work the authors used Microsoft Copilot in order to assist with translation, paraphrasing and language refinement of manuscript text derived from the original Dutch report. After using this tool, the authors reviewed and edited the content as needed and take full responsibility for the content of the published article.

## CRediT authorship contribution statement

**M. Kok:** Writing – review & editing, Writing – original draft, Visualization, Project administration, Methodology, Investigation, Formal analysis, Data curation, Conceptualization. **M.E.M. Den Ouden:** Writing – review & editing, Supervision. **I.M. Hertel:** Writing – review & editing, Validation. **J.P.M. van Bergen:** Writing – review & editing, Validation. **I. ten Haken:** Supervision, Methodology, Formal analysis. **C. Jaschinski:** Writing – review & editing, Supervision, Methodology.

## Ethics declaration

### Informed consent and patient details

Written informed consent to take part in the study and to publish the article has been obtained from all participants or their legal representatives. The privacy rights of participants have been observed.

### Studies in Human

This study was performed in compliance with relevant laws, regulatory frameworks and guidelines where the research took place.

The Medical Ethics Review Committee Oost-Nederland (METC Oost-Nederland) granted an exemption. The authors provided the following explanation: The study was submitted to the Medical Ethics Review Committee (METC) Oost-Nederland for assessment under the Dutch Medical Research Involving Human Subjects Act (WMO). The committee determined that participants were not subjected to procedures or behavioural requirements as defined under the WMO and concluded that the study was not subject to the WMO. Therefore, formal ethical review and approval were not required. In addition, the CMO Radboudumc confirmed that the study did not meet the criteria requiring review of non-WMO research.

## Declaration of competing interest

The authors declare that they have no known competing financial interests or personal relationships that could have appeared to influence the work reported in this paper.
